# Collagen hydrogel tube microbioreactors for cell and tissue manufacturing

**DOI:** 10.1088/1758-5090/ae2718

**Published:** 2025-12-12

**Authors:** Yakun Yang, Xinran Wu, Ying Pan, Yong Wang, Xiaojun Lian, Cheng Dong, Wansheng Liu, Shue Wang, Yuguo Lei

**Affiliations:** 1 Department of Biomedical Engineering, Pennsylvania State University, University Park, PA, United States of America; 2 Huck Institutes of Life Sciences, Pennsylvania State University, University Park, PA, United States of America; 3 Department of Animal Science, Pennsylvania State University, University Park, PA, United States of America; 4 Department of Chemistry, Chemical and Biomedical Engineering, University of New Haven, West Haven, CT, United States of America

**Keywords:** 3D cell culture, cell manufacturing, collagen hydrogel microtubes, microbioreactors

## Abstract

The large-scale production of mammalian cells, particularly stem cells for clinical applications, remains challenging with existing cell culture technologies such as two-dimensional cell culture flasks or three-dimensional stirred tank bioreactors. Current methods have issues such as excessive cell aggregation and significant shear stress-induced cell death, resulting in low cell yield, unacceptable batch-to-batch variation, high production costs, and difficulties in scaling up. We hypothesize that creating a cell-friendly microenvironment that has efficient mass transport and minimized shear stress can enhance cell culture efficiency. In this study, we developed a novel hydrogel tube microbioreactor using collagen proteins (ColTubes) to test this hypothesis. First, we designed an innovative micro-extruder for fabricating ColTubes loaded with cells. Our results show that collagen proteins form a dense and robust nanofiber network capable of shielding cells from hydrodynamic stress while maintaining cell mass below 400 *µ*m in diameter. The tube shell contains abundant nanopores that allow the cell culture medium to permeate and nourish the cells. Additionally, the collagen fibers serve as a substrate for cell adhesion. We show that ColTubes support high cell viability, rapid expansion, and impressive volumetric yields, offering substantial improvements over current methods. To our knowledge, ColTubes is a novel approach that has not been previously reported for cell manufacturing. ColTubes represents a scalable, cost-effective, and efficient solution for large-scale cell production.

## 1. Introduction

Mammalian cells have diverse applications. Stem cells, such as human pluripotent stem cells (hPSCs) and their derivatives, are crucial for regenerative medicine, disease modeling, drug screening, and toxicity testing [1]. Immune cells, such as T cells and natural killer cells, can be used to treat cancers [2–6]. HEK-293 and CHO cells are widely utilized for producing recombinant proteins and viruses [7–9]. All these applications require large numbers of cells [1]. For example, approximately 10⁹ cardiomyocytes (CMCs) or 10⁹ *β* cells are needed to treat a patient with myocardial infarction or Type 1 diabetes [10]. Engineering a human liver or heart requires approximately 10^10^ hepatocytes or CMCs [11] and screening a one-million-compound library needs 10^10^ cells [10].

However, culturing cells, especially stem cells, at large quantities is still very challenging [1, 10, 12]. *In vivo*, human cells live in a three-dimensional (3D) microenvironment that supports critical interactions between cells and the extracellular matrix (ECM), provides robust nutrient and oxygen supply, and minimizes hydrodynamic stress [13–17]. Current cell culture methods, however, often fail to replicate these conditions, resulting in low cell culture efficiencies and difficulties in scaling production. Two-dimensional (2D) cell culture systems, such as flasks, lack the complexity of *in vivo* 3D microenvironment and can only produce a limited number of cells per culture area. They are not suitable for large-scale cell production [1, 10, 12].

3D suspension culture technologies, such as stirred-tank and vertical wheel bioreactors, have been widely studied for scaling up cell production. However, they face challenges related to uncontrolled cell aggregation, particularly for stem cells with strong cell-cell interactions [16, 17]. In suspension cultures, cells form large aggregates. It is known that the transport of nutrients, oxygen, and growth factors to cells at the core of aggregates larger than 400 *µ*m becomes difficult, leading to slower proliferation, apoptosis, and undesired differentiation [10, 18]. Although agitation can reduce aggregation and improve mass transport, it can also introduce shear forces that negatively impact cell survival and growth [10, 19, 20]. Consequently, 3D suspension cultures often exhibit high cell death, slow growth rates, and low volumetric yields. For example, hPSCs typically expand less than 10-fold per passage to yield approximately 2.0 × 10^6^; cells ml^−1^. The cell mass occupies just 0.4% of the culture volume [21–23].

The complex hydrodynamic conditions in stirred-tank bioreactors—such as flow rate, shear stress, and chemical gradients—add further complications. These variables depend on multiple factors, including the bioreactor design, medium viscosity, and agitation speed, making precise control difficult [1, 10, 12, 19–24]. These lead to production variations. For instance, in recent studies to produce CMCs from hPSCs in stirred-tank bioreactors, the yields from three 100 ml batches varied from 40 to 100 million cells, with cardiomyocyte purity between 54% and 84%. Using a different hPSC line under the same conditions, the yields changed between 89 and 125 million cells, with purity varied from 28% to 88% [25, 26]. The hydrodynamic complexity also complicates scaling up. Culture conditions optimized at a small scale may not translate effectively to larger scales, requiring extensive re-optimization, which is time-consuming and costly. To our knowledge, the largest demonstrated scale for hPSC-derived CMCs using stirred-tank bioreactors is approximately 3 × 10⁹ cells per batch, sufficient to treat only three patients [27].

We hypothesize that creating an *in vivo*-like microenvironment—one that ensures efficient mass transport, minimizes shear stress, and supports 3D cell–cell and cell–matrix interactions—can significantly enhance cell culture efficiency [28]. We propose that hydrogel microtubes can be engineered to provide such an environment [28]. In our previous work, we demonstrated that culturing cells in hollow alginate hydrogel microtubes (AlgTubes) resulted in high cell viability, rapid proliferation, and exceptional volumetric yields, achieving up to 5 × 10⁸ hPSCs per milliliter—approximately 250 times higher than yields typically obtained in stirred-tank bioreactors [28–37]. These findings test our hypothesis and highlight the transformative potential of hydrogel tube microbioreactors for stem cell culture.

However, AlgTubes have certain limitations. For instance, they do not support the growth of anchor-dependent stem cells, as they lack adhesion points. Additionally, AlgTubes break frequently, leading to cell culture inconsistencies. Given the diversity of mammalian cell phenotypes and requirements, it is essential to develop hydrogel tubes with varied material properties. In this study, we introduce a second type of hydrogel tube made from collagen proteins (ColTubes). These tubes support cell adhesion to the inner surface and exhibit greater mechanical stability compared to AlgTubes. ColTubes also demonstrate high culture efficiency, comparable to that of alginate-based systems. We believe that the combined use of collagen and alginate hydrogel tube microbioreactors will enable scalable, cost-effective, and efficient cell production across a wide range of applications.

## 2. Methods

**Collagen extraction**. Rat tails were soaked in 70% ethanol to remove debris, and the skin was stripped away using a scalpel and forceps. Tendons were then collected and washed three times with PBS. Subsequently, they were sterilized in 70% ethanol for 1 h. Tendons were dissolved in 0.02 N acetic acid with continuous stirring at 4 °C for 48 h. The resulting solution was centrifuged at 10000 rpm for 60 min at 4 °C. The supernatant was dialyzed against 0.02 N acetic acid and collected as the collagen stock solution.

**Figure 1.**
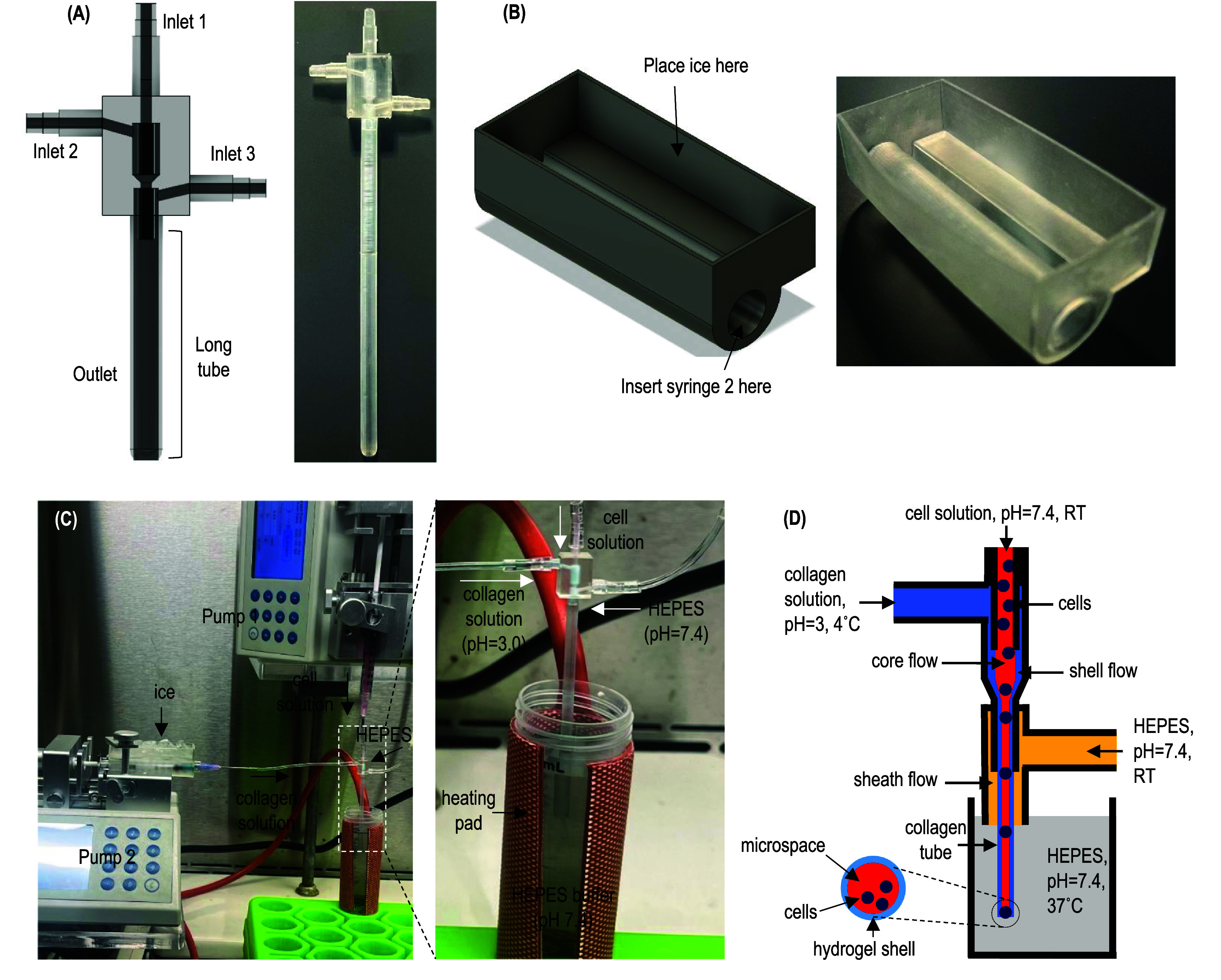
Fabricate collagen hydrogel tubes. (A) Design and print of the micro-extruder. (B) Design and print of the cooling box used for cooling collagen solution. (C) The setup for processing ColTubes, which includes three syringe pumps, the micro-extruder, the cooling box, a heating pad, and a HEPES buffer reservoir. (D) To fabricate ColTubes, a cell solution at room temperature (RT), an ice-cold collagen solution (pH = 3.0), and a HEPES buffer (RT, pH = 7.4) are pumped into inlet 1, 2, and 3 of the micro-extruder, respectively, to form coaxial core-shell-sheath laminar flows that are extruded into a heated HEPES buffer (37 °C, pH = 7.4). The shell collagen flow rapidly forms a hydrogel tube as a result of the combined pH neutralization and temperature increase.

**Fabricating collagen tubes.** A custom micro-extruder was designed using Fusion 360 (Autodesk) and fabricated using a stereolithography 3D printer (Form 3B+, Formlabs) (figure 1). The three inlets were connected to syringes mounted on syringe pumps (Fusion 200, Chemyx). Syringe 1, containing cells suspended in 1.5% methylcellulose, was connected to the core flow channel of the micro-extruder and pumped at 30 *μ*l min^−1^. Syringe 2, loaded with collagen solution, was placed in a custom ice box, connected to the shell flow channel, and pumped at 180 *μ*l min^−1^. Syringe 3 containing 50 mM HEPES buffer was attached to the sheath flow channel and pumped at 2 ml min^−1^. The extruder outlet was submerged in a 37 °C, 50 mM HEPES buffer maintained with a heating pad. Once the pumps were on, collagen tubes were continuously generated.

**Confocal microscopy**. To prepare bulk collagen hydrogel, the collagen solution was neutralized by adding a 10*x* neutralization buffer and incubated at 37 °C for 15 min. ATTO 488 NHS ester (Sigma) was used to label collagen fibers following the manufacturer’s guidelines. Briefly, Bulk collagen and ColTubes were reacted with 1 *µ*M ATTO 488 NHS ester in PBS for 15 min at room temperature, followed by three washes with PBS to remove excess dye. Confocal images were done using an Olympus FV3000 confocal microscope.

**Scanning electron microscope (SEM).** Bulk collagen hydrogel and ColTubes were dehydrated through an ethanol series (25%, 50%, 70%, 85%, 95%, and 100%) with 5 min at each concentration, followed by critical point drying using a Leica EM CPD300 Critical Point Dryer. Samples were then sputter-coated with a 4.5 nm iridium layer using a Leica EM ACE600 Sputter Coater. SEM images were acquired with a Zeiss SIGMA VP-FESEM SEM.

**Doping collagen tubes with laminins.** Recombinant Laminin 511 E8 fragments (iMatrix-511 SILK) were labeled with ATTO 594 NHS ester (Sigma) following the product instructions. The collagen solution was mixed with labeled laminins and processed into ColTubes to dope collagen tubes with laminins.

**Culturing hPSCs in ColTubes.** For a typical cell culture, H9 human embryonic stem cells (hESCs) (WA09, WiCell) loaded in 20 *µ*l ColTubes were suspended in 2 ml Essential 8 medium (Gibco) supplemented with 10 *μ*M Y-27632 (Sigma) in a 6-well plate and incubated at 37 °C with 5% CO₂ and 21% O₂. The medium was changed daily. To passage cells, the medium was removed, and ColTubes were dissolved by incubating with 0.2 mg ml^−1^ Collagenase P (Sigma) for 15 min. The cell mass was collected, treated with Accutase (StemCell Technologies) at 37 °C for 10 min, and dissociated into single cells for subsequent culture.

**Culturing hPSCs in 2D.** For 2D cell culture, hPSCs were cultured in 6-well plate coated with iMatrix-511 SILK in Essential 8 medium and incubated at 37 °C with 5% CO₂ and 21% O₂. The medium was refreshed daily. Cells were passaged using Accutase, with a 10 min incubation at 37 °C. 10 *μ*M Y-27632 was added for the first 24 h post-seeding and 2 h prior to passaging.

**hPSCs differentiation.** To induce differentiation of hPSCs into CMCs, the culture medium was switched from Essential 8 to E5 medium, consisting of DMEM/F12 (Corning), transferrin (Sigma), sodium selenite (Sigma), ascorbic acid (Sigma), and 1% Chemically defined lipid concentrate (Gibco). 7.5 *μ*M CHIR99021 (Selleck Chemicals) was added for the first 24 h to initiate mesoderm induction. From day 2 to day 5, 3 *μ*M IWR1 (Selleck chemicals) was added to promote cardiac lineage specification. 3 mg ml^−1^ heparin (Sigma) was included from day 1 to day 6 to support differentiation. On day 7, insulin (20 *μ*g ml^−1^; Sigma) was added to enhance cardiomyocyte maturation. To enrich the CMC population, the medium was replaced on day 11 with glucose-, glutamine-, and pyruvate-free DMEM (Gibco), supplemented with 5 mM lactate (Sigma), and maintained until day 18.

**EB differentiation.** For trilineage differentiation, hPSCs were directed into the three germ layers using the Human pluripotent stem cell functional identification kit (R&D Systems), following the product instructions.

**Culturing HEK293 in ColTubes.** For a typical cell culture, adherent HEK293 cells (#CRL-1573, ATCC) loaded in 20 *µ*l ColTubes were suspended in 2 ml DMEM supplemented with 10% fetal bovine serum (FBS) in a 6-well plate and incubated at 37 °C with 5% CO₂ and 21% O₂. Suspension 293 T 17SF cells (#ACS-4500, ATCC) in ColTubes were cultured in HyCellTransFx-H Medium (Cytiva). The medium was changed every three days.

**Flow Cytometry and Live/Dead Cell Staining.** LIVE/DEAD® Cell Viability Staining (Invitrogen) was performed according to the manufacturer’s instructions. For flow cytometry, single cells were fixed with 4% paraformaldehyde (PFA) at room temperature for 15 min, followed by incubation with PBS containing 0.1% Triton X-100, 0.5% BSA, and primary antibodies from the human pluripotent stem cell marker antibody panel (R&D Systems) at 4 °C overnight. After thorough washing, secondary antibodies (Biotium) were added and incubated for 2 h at room temperature. Cells were washed three times with PBS containing 0.5% BSA before analysis using the Attune® NxT™ Acoustic Focusing Cytometer. Data analysis was conducted using FlowJo software.

**Fabricating seminiferous tubules.** TM3 Leydig cells (#CRL-1714, ATCC) and TM4 Sertoli cells (#CRL-1715, ATCC) were cultured according to ATCC instructions. Briefly, cells were maintained in DMEM/F12 supplemented with 5% horse serum and 2.5% FBS at 37 °C and 5% CO₂ with media changes every 2–3 d. TM3 cells were labeled with CellTrace™ CFSE, and TM4 cells were labeled with CellTrace™ Far Red (Fisher) following product instructions. TM4 and TM3 cells were mixed into the core and shell flow to process ColTubes.

**Statistical analysis.** Data was analyzed using GraphPad Prism 8 statistical software and shown as mean ± standard error of the mean. *P* values were calculated using one-way analysis of variance for comparisons among three or more groups, unpaired two-tailed t-tests for comparisons between two groups, and one-sample two-tailed t-test for comparison between experimental and theoretical values. The significance levels are indicated by p-value, ns: not significant; *: *p* < 0.05, **: *p* < 0.01, ***: *p* < 0.001.

## 3. Results

### 3.1. The Extrusion System for Processing Collagen Hydrogel Tubes (ColTubes)

A novel micro-extruder with three inlets and one outlet was designed for processing ColTubes (figure 1(A)). The extruder was fabricated using a Form 3B+ printer and clear resin. A cooling box was also designed and fabricated based on 3D printing (figure 1(B)). The ColTube processing setup consists of three syringe pumps, the micro-extruder, the cooling box, a heating pad, and a conical tube or container containing HEPES buffer (figure 1(C)). The three syringes contain the following solutions: (1) a cell solution at room temperature (RT, pH = 7.4), (2) an ice-cold collagen solution (pH = 3.0), and (3) a HEPES buffer (RT, pH = 7.4). The cooling box has a channel for holding syringe 2 (figure 1(B)). Ice is loaded into the box to maintain the collagen solution in syringe 2 at a temperature below 4 °C. The collagen is dissolved in 0.02 N acetic acid to have a pH of 3.0, which, along with the low temperature, prevents premature collagen gelation.

To fabricate ColTubes, the three solutions are pumped into inlets 1, 2, and 3 of the micro-extruder, respectively, forming coaxial core-shell-sheath laminar flows that are extruded into the heated HEPES buffer (37 °C, pH = 7.4) (figure 1(D)). The core, shell, and sheath flow contain the cells, collagen proteins, and HEPES buffer, respectively. The collagen solution is neutralized by the core solution and the HEPES buffer in both the sheath flow and the conical tube. Additionally, the collagen solution is rapidly heated by the HEPES buffer in the conical tube, forming a stable collagen tube.

In summary, our innovative micro-extruder design, combined with the cooling box and heating pad setup, enables the formation of stable core-shell-sheath coaxial laminar flows while achieving rapid pH and temperature changes in the collagen flow. These allow the rapid formation of microscale ColTubes for cell culture. To the best of our knowledge, there are no existing scalable technologies that can rapidly process microscale collagen hydrogel tubes without compromising cell viability.

### 3.2. Engineering Principles and Control of ColTube Dimensions

**Figure 2.**
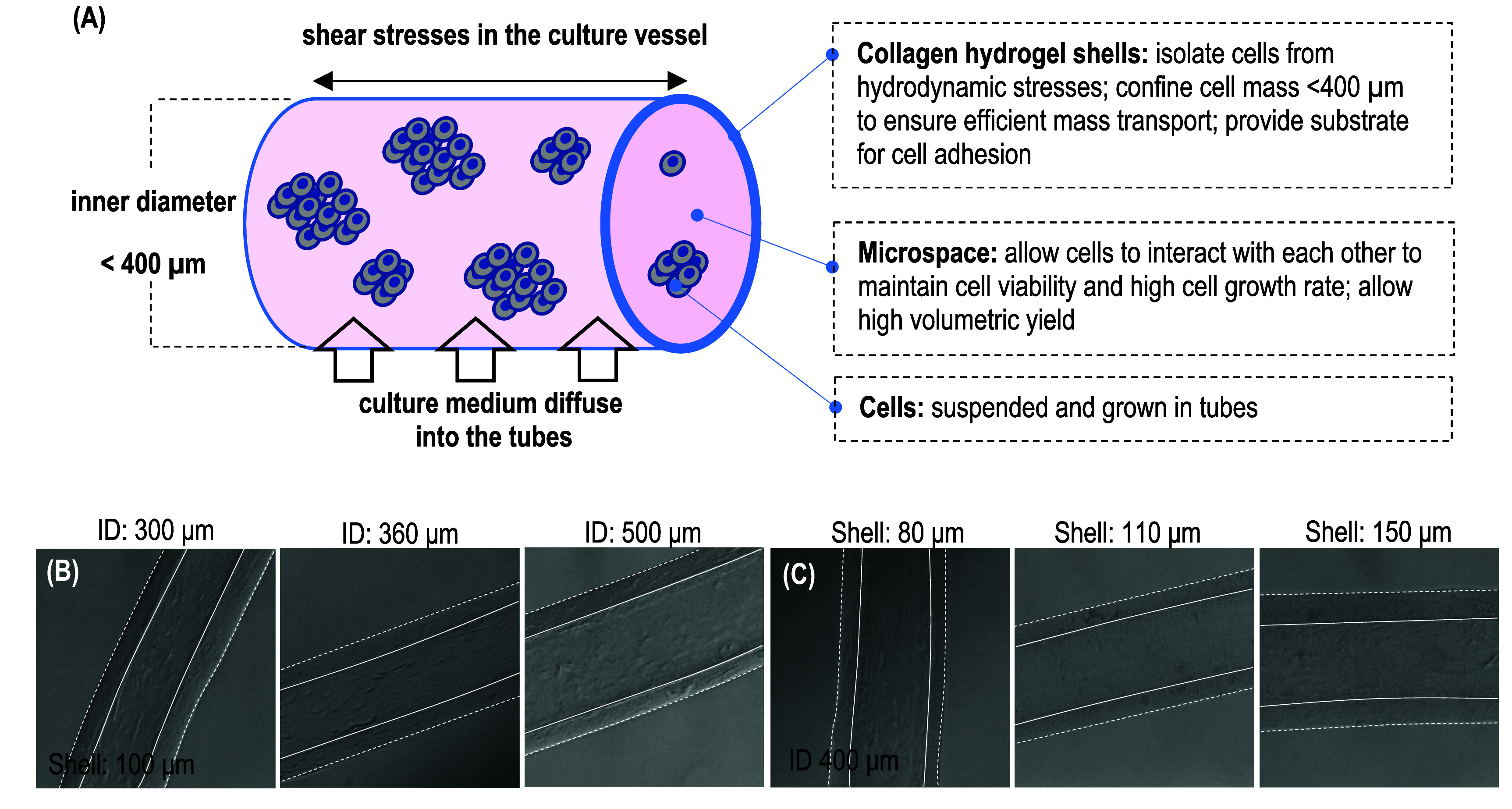
Engineering principles. (A) Cells are grown within hydrogel tubes suspended in a culture medium. The tubes protect cells from hydrodynamic stresses, restrict the cell mass to a radial diameter of <400 *µ*m, provide free space for cell growth, and serve as a substrate for cell adhesion. The cell culture medium enters the tubes through the nanopores in the tube wall. (B) The inner diameter (ID) is varied from 300 to 500 *µ*m, while maintaining a constant shell thickness of ∼100 *µ*m. (C) The shell thickness is adjusted from 80 to 150 *µ*m, while keeping the ID constant at ∼400 *µ*m.

In our system, cells are cultured within ColTubes suspended in the culture medium. ColTubes are engineered to address the limitations of conventional cell culture technologies by providing a physiologically relevant microenvironment (figure 2(A)). The hydrogel tubes serve as physical barriers that prevent excessive cell aggregation. Their highly porous shell allows nutrients and waste to freely diffuse, ensuring that the medium can reach the cells inside the tube. The tube has an inner diameter of less than 400 *µ*m, meaning that even when fully filled with cells, the resulting cell mass remains below the diffusion limit of approximately 500 *µ*m observed in human tissues. This design supports efficient nutrient and waste exchange throughout the culture period.

Additionally, the hydrogel tube shields cells from hydrodynamic shear stress within the culture vessel. The confined microspaces created by the tubes promote efficient cell–cell and cell–matrix interactions, enabling robust cell expansion. Our previous studies have shown that these microspaces are critical for achieving high cell viability, proliferation, and volumetric yield [28, 31]. Cells embedded directly in bulk hydrogel scaffolds exhibit significantly lower viability and growth rate and volumetric yield [28, 31]. Finally, the collagen fibers forming the tube walls provide a biological substrate for cell adhesion, which is essential for the culture of anchorage-dependent cells.

The ColTube dimension can be controlled by adjusting the core, shell, and sheath stream flow rates. The HEPES sheath flow not only neutralizes the acidic collagen shell solution but also acts as a hydrodynamic focusing mechanism to control the ColTube diameter. Increasing the sheath flow rate reduces the diameter. The shell thickness can be adjusted by changing the collagen shell flow rate—a lower shell flow rate results in a thinner tube wall. Figures 2(B) and (C) show ColTubes with varied diameters and shell thicknesses. A preliminary mathematical equation for predicting tube geometry has been built (figure S1). Future work should systematically characterize the fluidic dynamics in the extruder to make the model more precise.

### 3.3. Nanostructures of ColTubes

**Figure 3.**
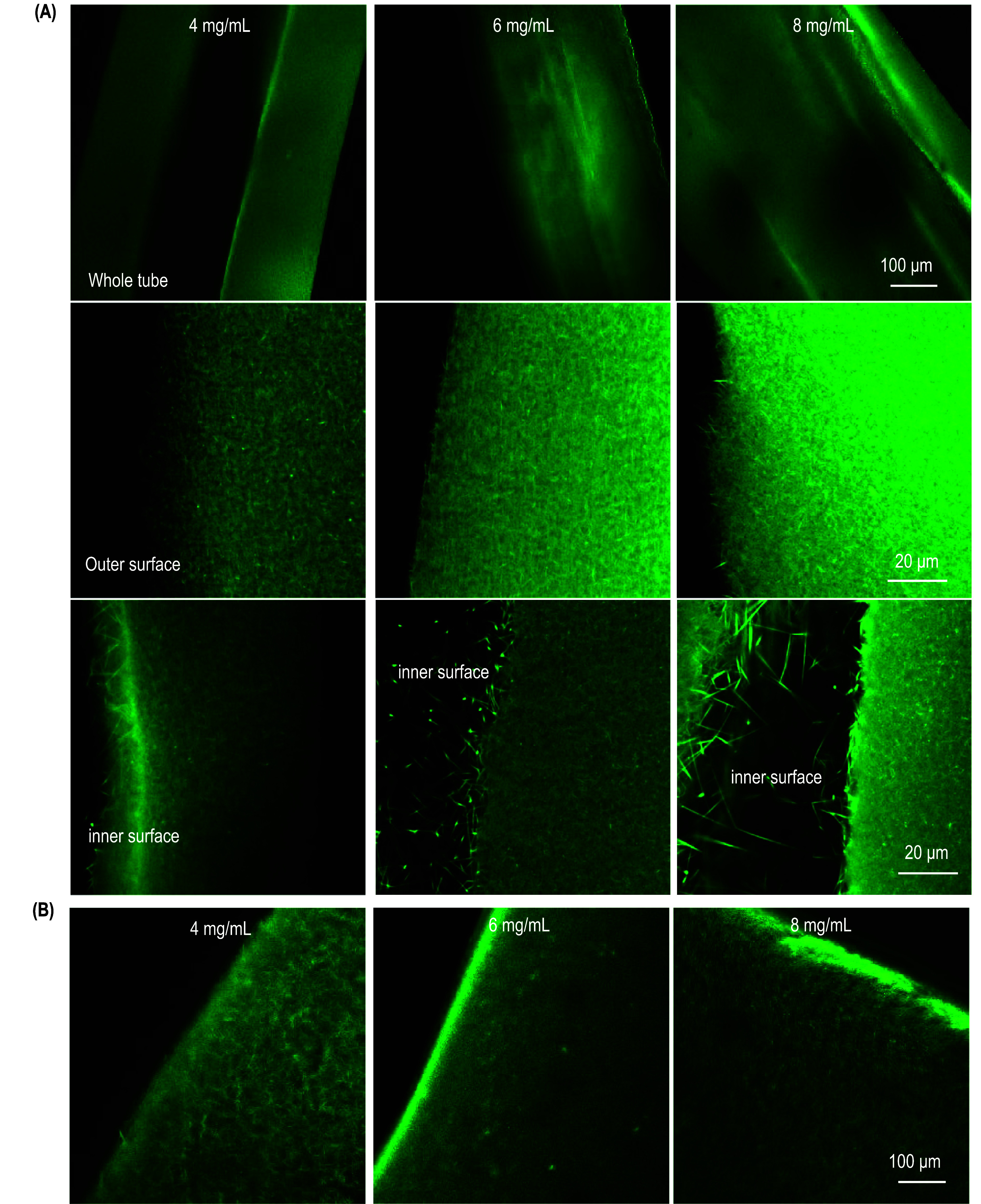
Nanostructures of ColTubes and Bulk Collagen Hydrogels. (A) ColTubes and (B) bulk collagen hydrogels were fabricated using 4-, 6-, and 8 mg ml^−1^ collagen. Nanostructures were imaged using confocal microscopy. For ColTubes, images include the entire tube and the outer and inner surfaces.

ColTubes were labeled with ATTO 488 NHS ester and imaged using a confocal microscope (figure 3(A)). Bulk collagen hydrogels were also prepared and imaged for comparison (figure 3(B)). Collagen proteins formed a dense nanofiber network in ColTubes, similar to these in the bulk hydrogel, indicating the gelation mechanism in ColTubes and Bulk hydrogel were similar. The nanostructures of the outer surface and the tube shell were similar, while some loose collagen nanofibers protruded inward at the inner surface. The collagen concentration in the tested range (3–8 mg ml^−1^) had minimal influence on the nanofiber diameter, length, orientation, and the nanofiber network’s porosity and pore size.

**Figure 4.**
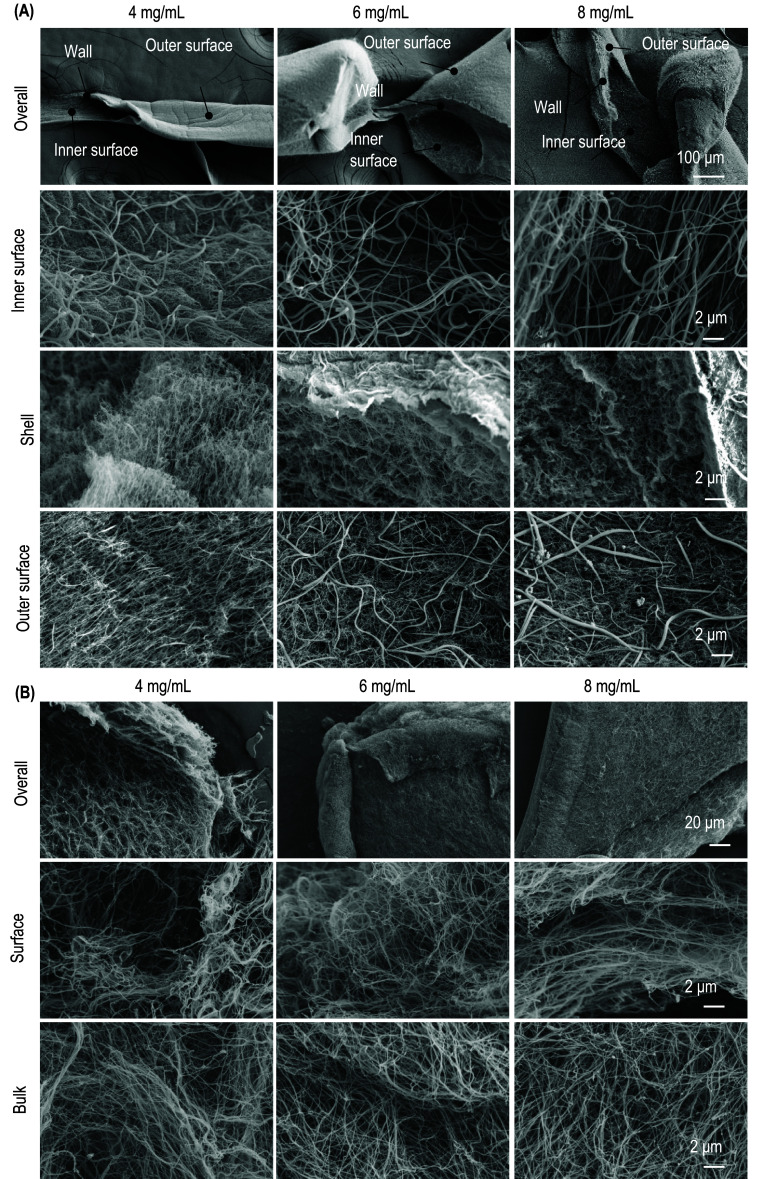
Nanostructures of ColTubes and bulk collagen hydrogels. (A) ColTubes and (B) bulk collagen hydrogels were prepared using 4-, 6-, and 8 mg ml^−1^ collagen. Nanostructures were imaged using scanning electron microscopy. For ColTubes (A), images include the entire tube, inner surface, shell, and outer surface. For bulk hydrogels (B), both bulk and surface structures are shown.

We used SEM to investigate detailed nanostructures. ColTubes were fixed with PFA and dehydrated through an ethanol series, followed by metal sputtering and SEM imaging. The whole tube, inner surface, outer surface, and shell were imaged (figure 4(A)). Bulk collagen hydrogels were used for comparison (figure 4(B)). Collagen nanofibers formed a dense network within the ColTube shell. Nanofibers were less densely packed at the inner and outer surfaces, with some protruding inward at the inner surface, consistent with confocal microscopy observations. No notable differences in nanostructures were observed between ColTubes and Bulk collagen hydrogels. Moreover, the collagen concentration had a minor impact on the hydrogel structures.

**Figure 5.**
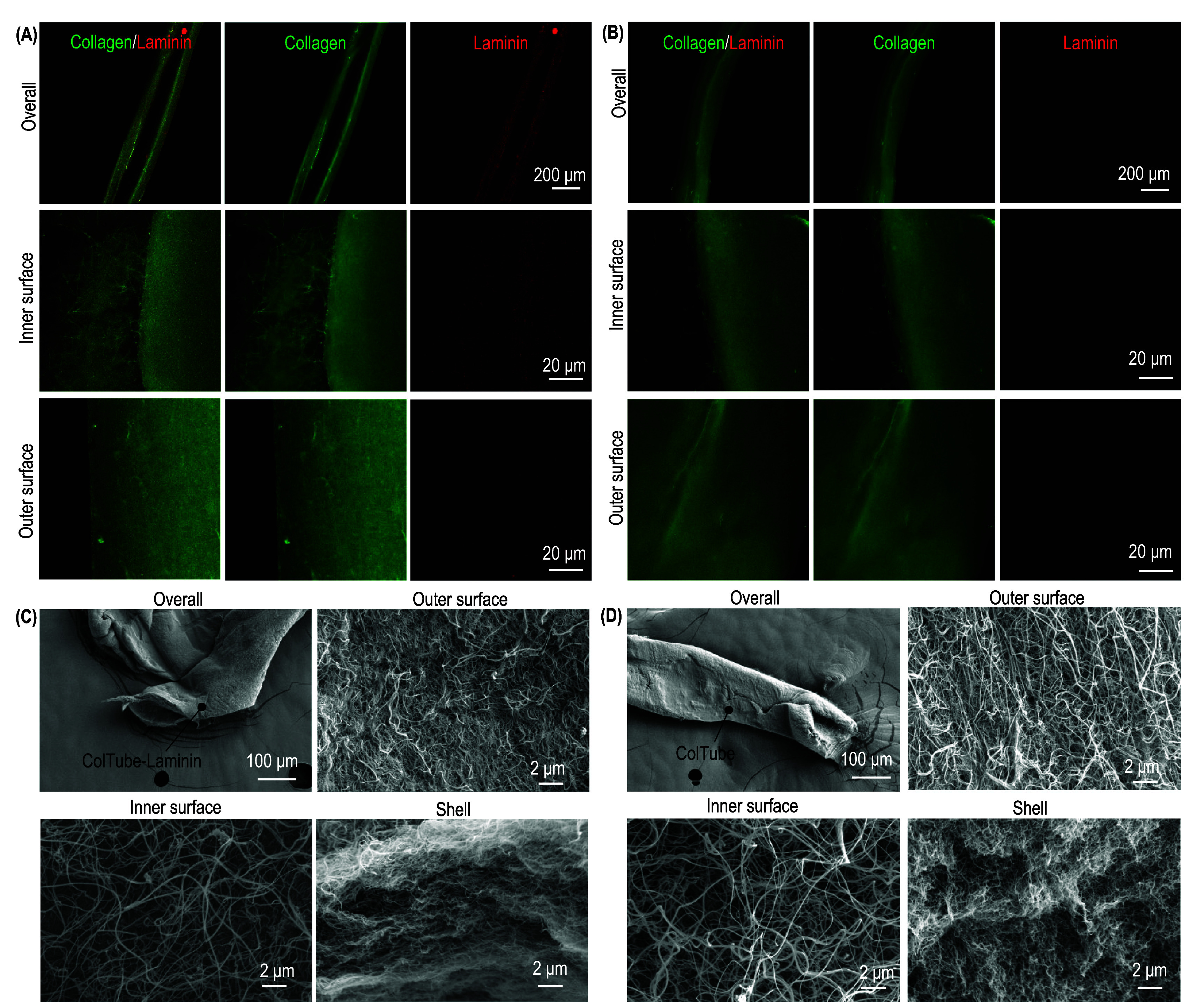
ColTubes doped with laminins. Confocal microscopy and SEM images of ColTubes with or without laminin doping: (A) Confocal images of the whole tube, inner surface, and outer surface of ColTubes doped with laminin. (B) Confocal images of ColTubes without laminin doping. (C) SEM images showing the whole tube, outer surface, inner surface, and shell of ColTubes doped with laminin. (D) SEM images of ColTubes without laminin doping.

Although most mammalian cells can adhere to collagen fibers, certain stem cells require specific ECM proteins for adhesion. For example, hPSCs are often cultured on plates coated with laminins. To test whether ECM proteins can be incorporated and maintained in ColTubes, we labeled recombinant Laminin 511 protein with Alexa Fluor 594 NHS Ester (emitting red fluorescence). We mixed it with collagen to process ColTubes. The tubes were soaked in PBS for three days to wash away soluble laminins. The tubes were subsequently fixed with 4% PFA and labeled with Alexa Fluor™ 488 NHS Ester to visualize collagen and laminin in green fluorescence. Images showed that laminins were successfully incorporated and remained in ColTubes (figure 5(A)). Control ColTubes without laminins did not exhibit any red fluorescence signal (figure 5(B)). SEM analysis found that laminin doping did not alter ColTube nanostructures (figures 5(C) and (D)). Our data showed that adding laminin could enhance the adhesion of hPSCs to ColTubes (figure S2). In short, the properties of ColTubes can be tailored by adding other ECM proteins. Future work can systematically study how different ECM proteins affect stem cell culture.

### 3.4. Culturing Cells in ColTubes

**Figure 6.**
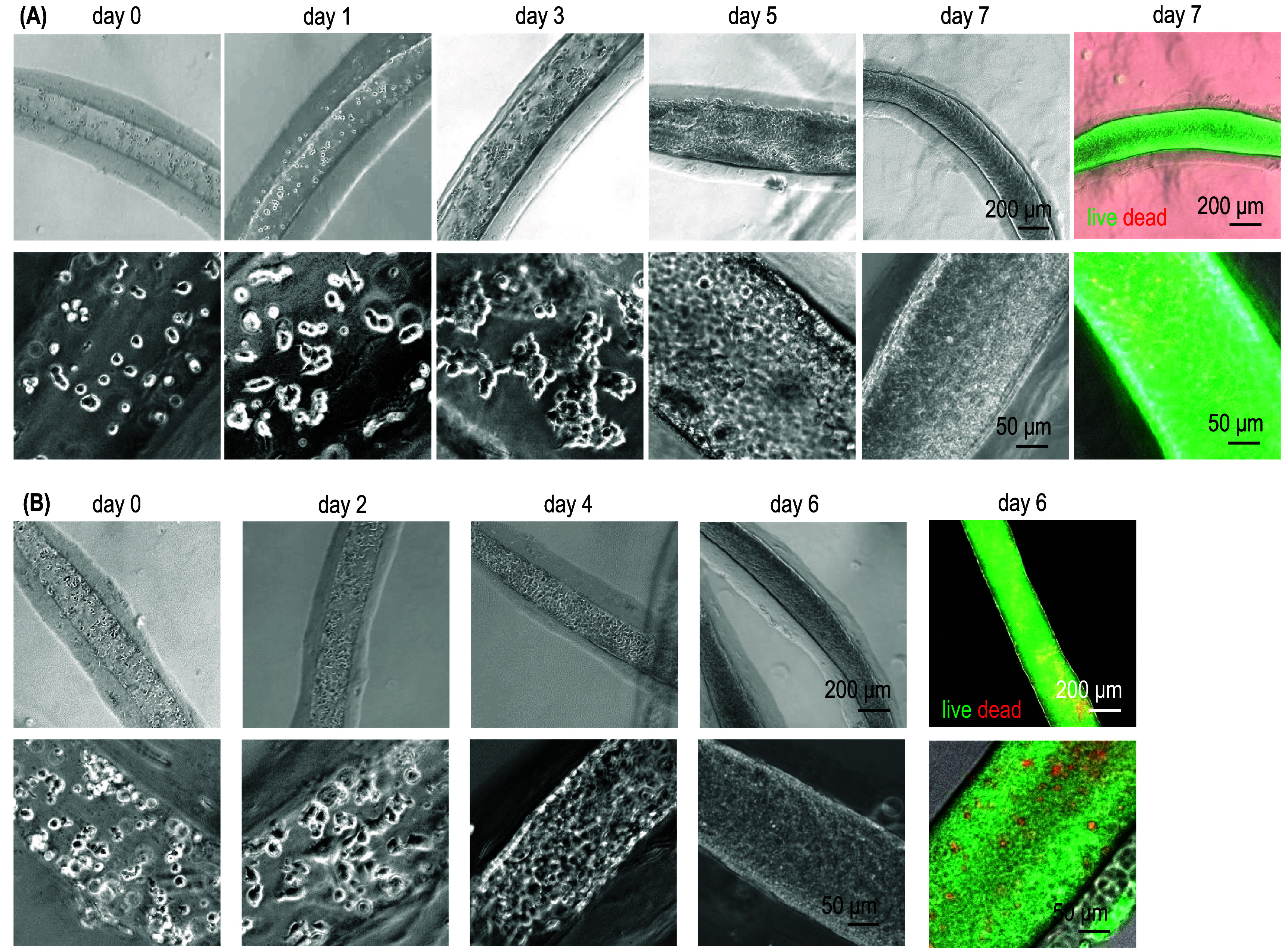
Culturing Adherent HEK 293 T Cells in ColTubes. Phase-contrast and Live/Dead staining images of adherent HEK 293 cells (A) and suspension 293 T 17SF cells (B) in ColTubes.

HEK293 cells are widely utilized for producing protein and viral therapeutics. The original adherent HEK293 cells have also been adapted to suspension culture. We tested the suitability of ColTubes for culturing adherent (figure 6(A)) and suspension (figure 6(B)) HEK293 cells. After 24 h, both cells adhered to the inner surface and expanded to form colonies attached to the tubes. Over time, cells proliferated and filled the entire tube. Live/Dead cell staining showed that most cells remained viable, and we could harvest >3 × 10^8^ cells from one milliliter of microspaces. When the suspension HEK293 cells were directly embedded in collagen hydrogel microfibers, cells had significantly higher death and much less volumetric yield (figure S3), consistent with our previous studies using AlgTubes [28, 31].

**Figure 7.**
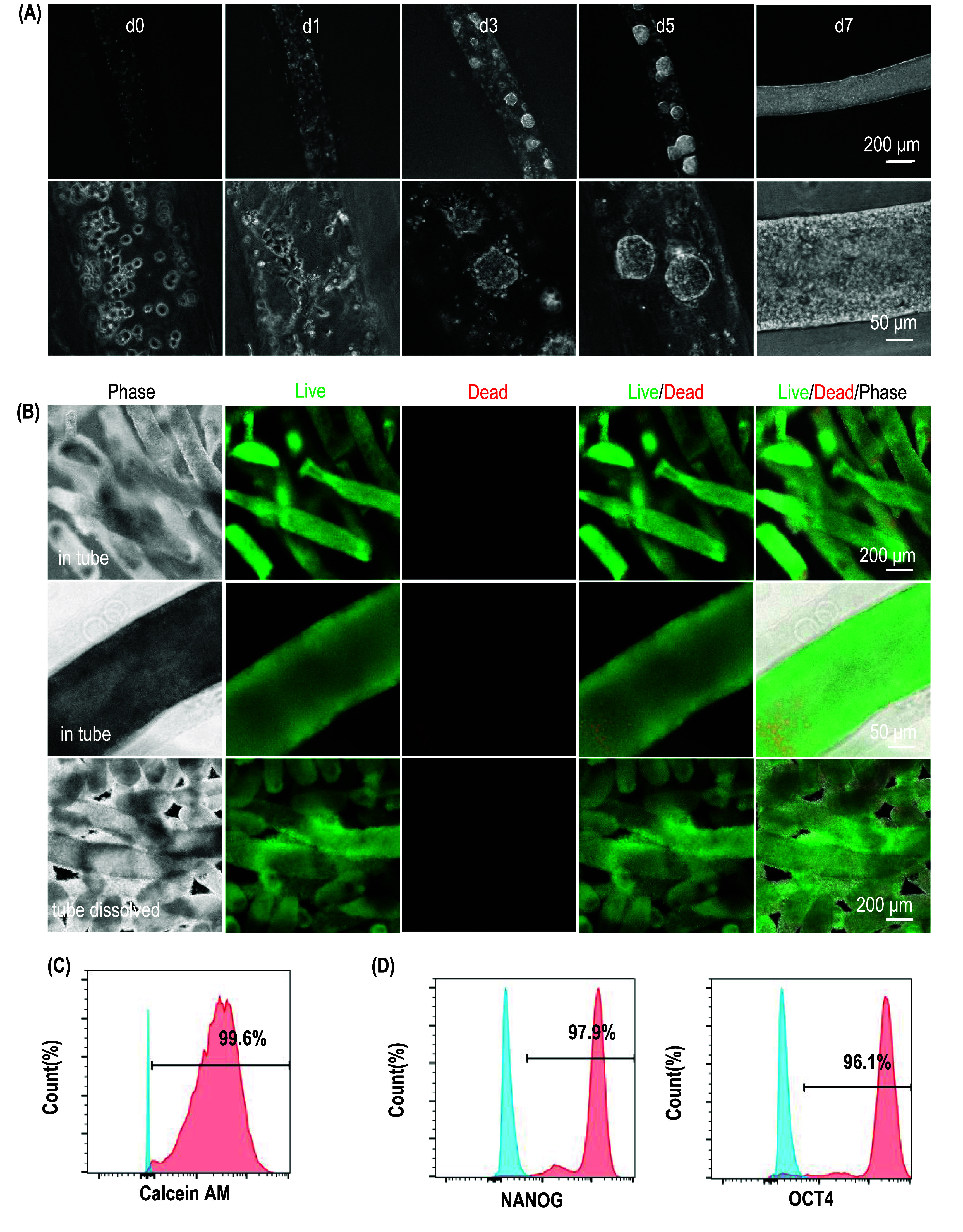
Culturing human embryonic stem cells in ColTubes. (A) Phase-contrast images of H9 hESCs cultured in ColTubes at days 0, 1, 3, 5, and 7. (B) Live/Dead staining of H9 cells inside the tubes and released from the tubes on day 7. (C) Flow cytometry analysis shows that 99.6% of cells harvested on day 7 are Calcein AM-positive (live cells). (D) Flow cytometry analysis confirms that majority cells on day 7 express pluripotency markers OCT4 and Nanog.

hPSCs, including hESCs and induced pluripotent stem cells, are ideal starting cells to prepare various human cells for treating diseases due to their unlimited proliferation capability and ability to differentiate into all human cell types. We cultured H9 hESCs in ColTubes. Single cells adhered to the inner surface within 24 h. By day 3, cells formed small colonies attached to the tube’s inner surface, which grew into spheroids by day 5. By day 7, cells filled most of the tubes (figure 7(A)). Live/Dead staining of cells before and after release from ColTubes showed minimal cell death (figure 7(B)). Flow cytometry confirmed that 99.6% of cells were viable (figure 7(C)). 97.9% and 96.1% of the cells expressed pluripotency markers, Nanog and Oct4, respectively, indicating that cells retained their pluripotency after culturing in ColTubes (figure 7(D)). Using ColTubes, we achieved over 4.5 × 10⁸ cells per milliliter of microspaces yield, which is approximately two orders of magnitude higher than what was achieved with stirred-tank bioreactors in our previous studies [1, 21, 28]. These cells had the typical pluripotent stem cell morphology when cultured in 2D flask (figure S4).

**Figure 8.**
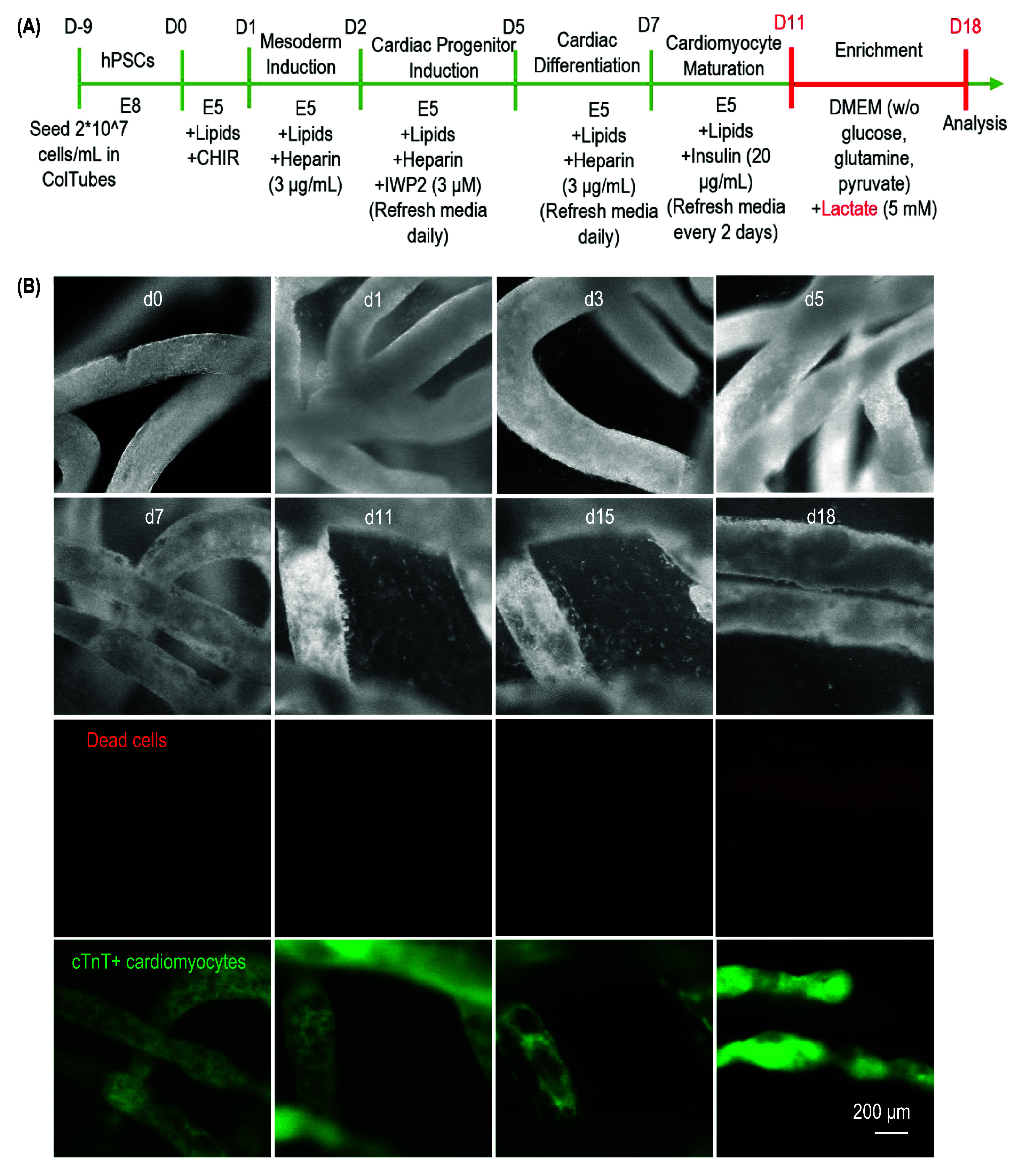
Differentiating H9 hESCs into Cardiomyocytes in ColTubes. (A) The cardiomyocyte production protocol. H9 hESCs are processed into ColTubes and expanded in E8 medium, followed by mesoderm induction for 1 d, cardiomyocyte differentiation from days 2–11, and metabolic enrichment from days 11–18. (B) Phase-contrast and fluorescent images of cells in ColTubes on days 0, 1, 3, 5, 7, 11, 15, and 18. Cardiomyocytes are cTnT-positive.

We tested whether hPSCs could be differentiated into functional cells in ColTubes. H9 hESCs carrying a GFP reporter under the cTnT promoter were expanded in ColTubes in the E8 medium for 7 d. Without cell passaging, the expansion medium was switched to a mesoderm induction medium to differentiate hESCs into mesoderm progenitors. On day 2, the medium was changed to cardiac progenitor differentiation medium, followed by cardiomyocyte differentiation medium on day 7. Between days 11 and 18, cells were cultured in a metabolic enrichment medium (figure 8(A)). Most cells remained viable, and over 90% of the final cells were cTnT+ CMCs (figure 8(B)). We produced over 3 × 10^8^ cells per milliliter of hydrogel tubes. In short, we have shown that ColTubes can be used to culture cells with high cell viability, growth rate and volumetric yield. Future work should systematically study if cells cultured in ColTubes have similar phenotypes to current cell culture methods.

### 3.5. ColTubes Exhibit Fewer Cell Leakage Events Compared to AlgTubes

**Figure 9.**
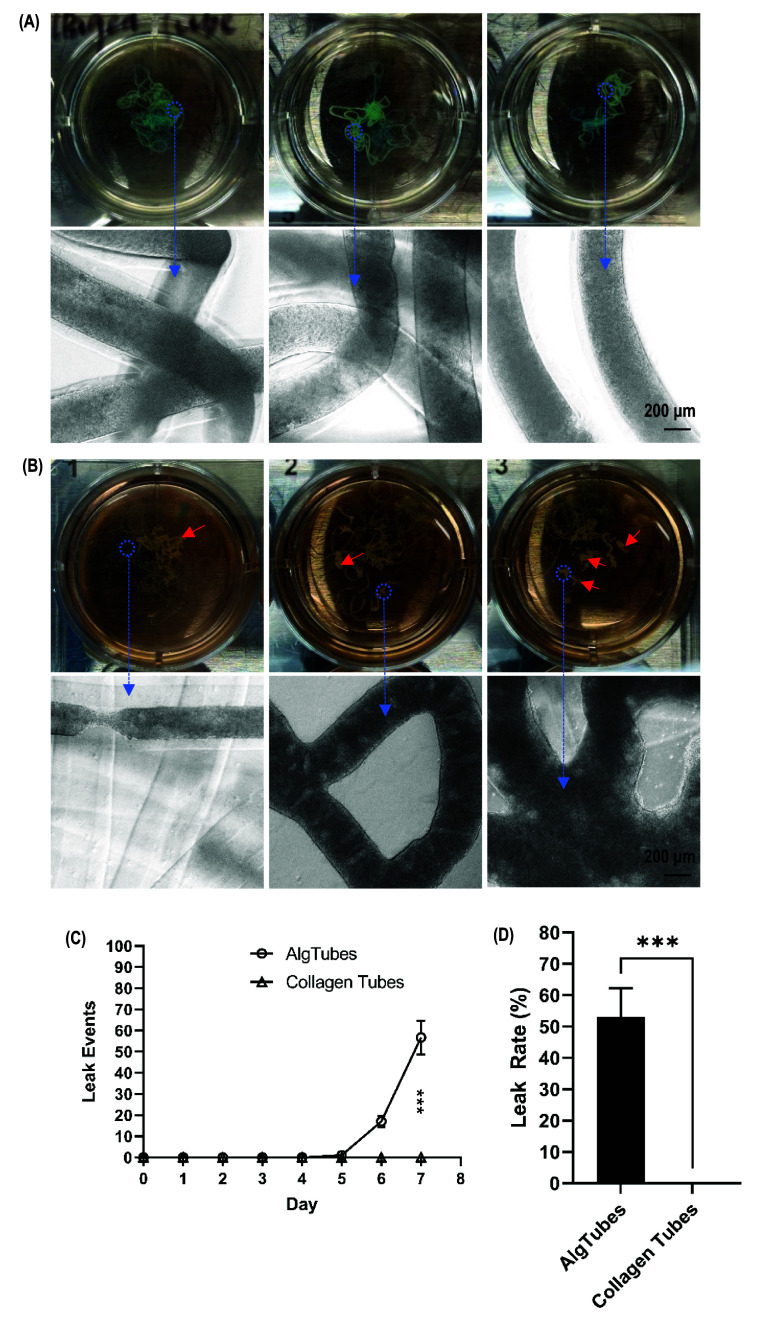
ColTubes exhibit fewer cell leakage events compared to algtubes. (A) Images showing no cell leakage from ColTubes. (B) Images showing significant cell leakage from AlgTubes, with large cell aggregates forming (red arrow). (C) Quantification of cell leakage events over time for AlgTubes and ColTubes. (D) Leak rate comparison between AlgTubes and ColTubes. Leak rate is defined as the number of cells leaked into the medium divided by the total number of cells in the well.

Previously, we used alginate-based hydrogel tubes for cell culture. However, AlgTubes break occasionally, leading to cell leakage and culture variation. As shown in figure 9(B), cell leakage was observed using AlgTubes. Leaked cells formed large aggregates. In contrast, no cell leakage was observed in ColTubes (figure 9(A)). Quantification of leakage events (figure 9(C)) and the percentage of cells leaked into the medium on day 7 (figure 9(D)) confirmed that ColTubes exhibited no leakage events, whereas AlgTubes did.

### 3.6. Building Tubular Tissue Models with ColTubes

**Figure 10.**
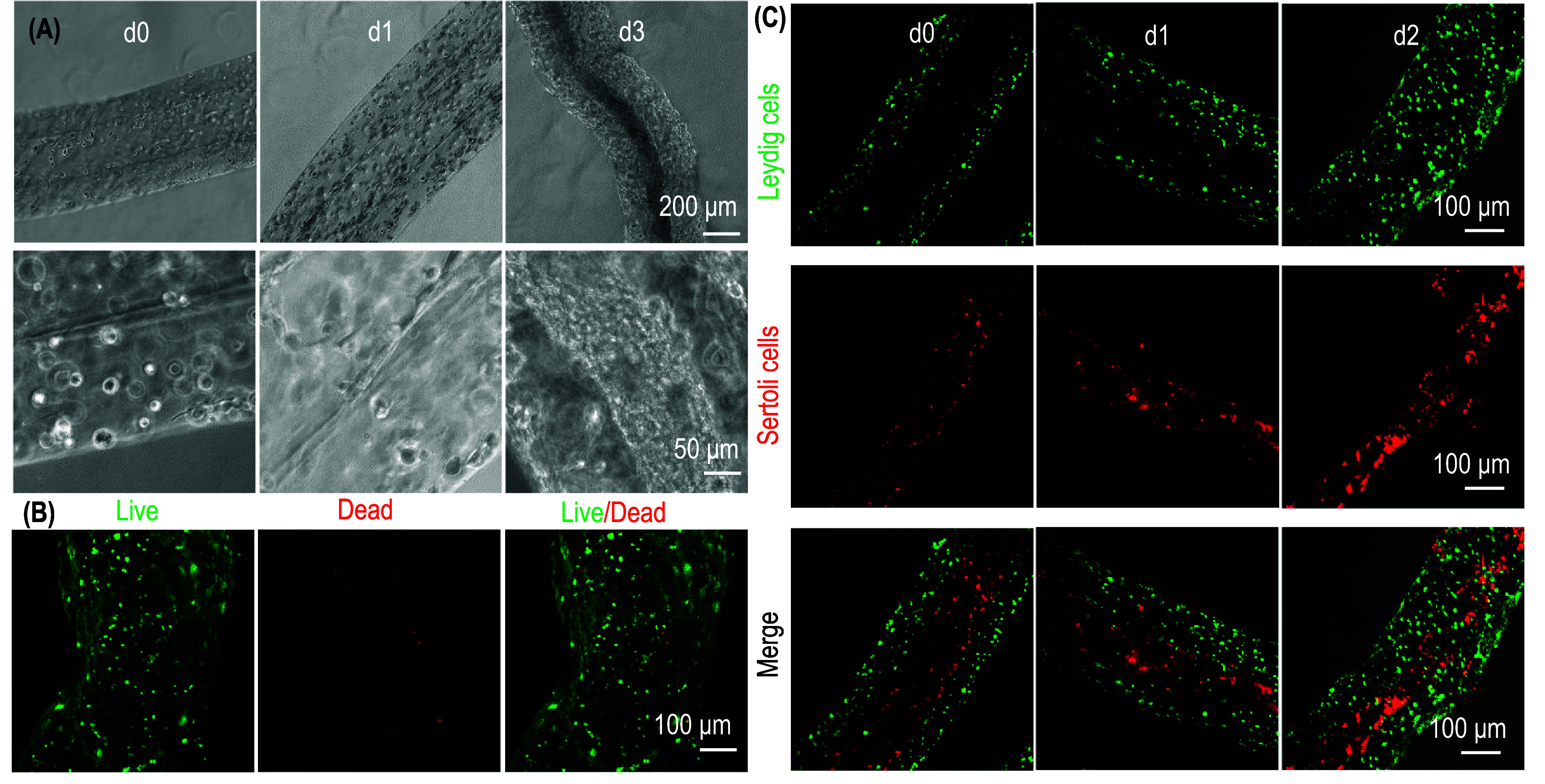
Fabricating seminiferous tubules with coltubes. (A) Phase-contrast images of coltubes containing leydig cells in the collagen shell and sertoli cells inside the tube at days 0, 1, and 3. (B) Live/Dead staining of the tubules on day 5 shows minimal cell death. (C) Confocal images showing leydig cells (green) in the collagen shell and sertoli cells (red) inside the tube.

In addition to cell production, ColTubes can be used to fabricate tissues for therapeutic applications and disease modeling. Human sperm is produced in seminiferous tubules within the testis, where Sertoli cells form a monolayer lining the inner surface. Leydig cells and other minor cell types reside in the surrounding interstitial tissue. To mimic this structure, we mixed Leydig cells with collagen to form the shell flow and suspended Sertoli cells in the core flow. The resulting ColTubes successfully contained Leydig cells within the tube shell and Sertoli cells on the inner surface, resembling the *in vivo* seminiferous tubules (figure 10).

In summary, our results show that ColTube is a versatile platform for cell expansion, differentiation, and tissue modeling. ColTubes exhibit excellent mechanical properties, preventing cell leakage and ensuring culture integrity. Moreover, coltubes allow cells to adhere, making coltubes suitable for culturing both adherent and suspension cells.

## 4. Discussion

Current 2D and 3D cell culture methods face significant challenges in achieving robust and cost-effective large-scale cell production, particularly for stem cells used in clinical applications [38–40]. Key issues include low cell yields, limited scalability, high costs, and significant culture variability. The fundamental problem is that these methods provide a cellular microenvironment that differs substantially from the natural *in vivo* 3D microenvironment. It is hypothesized that replicating a biomimetic microenvironment can address these limitations and substantially improve cell culture efficiency [28, 31].

We proposed that hydrogel tube microbioreactors are a promising approach for creating a cell-friendly microenvironment [28, 31]. To evaluate this approach, we previously developed a method to fabricate hydrogel tubes using alginate polymers [28–37]. Alginates are widely available, inexpensive, biocompatible, and have been safely used in clinical applications. These polymers can rapidly crosslink with calcium ions to produce large quantities of AlgTubes through an extrusion process. After culture, AlgTubes can be dissolved with an EDTA solution to harvest the product. The tubes are also transparent, facilitating real-time observation of cell growth [28–37].

Using AlgTubes, we successfully cultured hPSCs over multiple passages with high consistency while maintaining pluripotency and chromosomal stability [28]. The cultures showed excellent viability, expansion, and yields (∼5 × 10⁸ cells ml^−1^ of microspace), surpassing stirred-tank bioreactors by approximately 250-fold. Up to 4200-fold expansion per passage was achieved, far exceeding conventional 3D suspension cultures, which typically is less than 10-fold per passage. Additionally, hPSCs could be differentiated into various tissue cells, such as endothelial cells [29, 32], vascular smooth muscle cells [35], neural stem cells [30], and neurons [33] in AlgTubes, all achieving yields of 5 × 10⁸ cells ml^−1^. Adult cell types such as T cells could also be cultured in AlgTubes [34]. We showed that the microspaces were critical as cells directly embedded in hydrogel scaffold had much less cell growth and volumetric yield [28, 31]. These results highlight the transformative potential of AlgTubes for high-efficiency, large-scale cell production.

However, given the diverse phenotypes of mammalian cells and their varying requirements for growth environments and substrates, AlgTubes alone are insufficient to support all cell types. Most mammalian cells do not express receptors for alginates and therefore do not adhere to AlgTubes. The survival and proliferation of many cell types, such as mesenchymal stem cells and endothelial cells, require adhesion to a substrate. AlgTubes are only suitable for culturing anchor-independent cells, such as hPSCs and T cells. Additionally, AlgTubes occasionally break, leading to cell leakage and culture variability (figure 9). Furthermore, some cell culture media contain chelators that can extract Ca^2+^ from the alginate hydrogel, causing AlgTubes to dissolve. There is a need to fabricate hydrogel microtubes using other materials.

In this study, we successfully developed a second type of hydrogel tube using collagen proteins. Current technologies are unable to efficiently process collagen proteins into stable microtubes. Previous research has shown that collagen solutions at pH 3.0 can form hydrogels when extruded into a buffer at pH 7.4 [41]. However, fabricating stable microtubes is significantly more complex than producing solid hydrogels and has not been achieved previously. By integrating a cooling box, a three-flow micro-extruder, a heating pad, and a buffer exchange system (figure 1), we successfully developed a rapid method to produce ColTubes.

The resulting ColTubes had a dense nanofiber network that was stable in most cell culture media. They exhibited remarkable durability, with no breakages occurring during cell culture, thereby preventing cell leakage and ensuring consistent production (figure 9(A)). Furthermore, most cells express receptors for collagen, allowing them to adhere naturally (figures 6 and 7). Additionally, collagen contains binding domains for other ECM proteins, such as fibronectin and laminin. These proteins can be incorporated into ColTubes to support the growth of specialized cell types (figure 5). Our results showed that cells grew in ColTubes (figures 6–8) as efficiently as they did in AlgTubes [28–37].

Beyond cell culture, we also showed that ColTubes could be used to construct tissue models. The human body is rich in microscale tubular tissues, such as blood capillaries, lymphatic vessels, seminiferous tubules, and milk ducts. Many cancers, including various carcinomas, arise from epithelial transformations in tubular structures. In these tissues, endothelial or epithelial cells line the inner surfaces of the tubes, while stromal cells form the shells and interstitial regions between tubes. ColTubes offer a platform for creating analogous tubular tissue models by placing endothelial or epithelial cells on the inner surfaces and stromal or interstitial cells within the tube shell (figure 10). These engineered tissues have broad applications, including disease modeling, drug screening, and regenerative medicine.

ColTube is a valuable tool for academic laboratories and biotechnology companies. ColTubes will enable labs to perform experiments that need large numbers of cells. For instance, 3D printing a human heart would need at least 1 × 10^9^ cells, which requires a ∼1 l stirred tank bioreactor to produce—a task that is difficult to complete in most research labs. The same number of cells can be made with 2 ml ColTubes, which can be readily done in a research lab, as shown in a proof-of-concept study in our lab (figure S5).

ColTubes also have the potential to accelerate translational research. Taking the development of hPSC-derived CMCs for myocardial infarction treatment as an example [42], early-stage research typically uses 2D flasks to generate cells for testing efficacy and safety in rodents [43]. Large-scale 3D suspension culture is then required to produce sufficient cells for large-animal studies and clinical trials [44, 45]. Developing such a 3D bioprocess requires significant time and investment [46]. ColTubes offer a scalable platform suitable for all stages of the drug development pipeline. For instance, 2 ml of ColTubes can produce 10⁹ cells for small-animal studies, while 200 ml can yield 10^11^ cells for large-animal studies and clinical trials. Further scaling up for industrial-scale production can be readily achieved. By eliminating the need for multiple bioprocess transitions, ColTubes can significantly reduce the time, effort, and costs associated with developing cell-based therapeutics.

The high growth rate and cell density of ColTubes have a significant impact on industrial-scale cell production. For example, producing 10^12^ hPSC-derived CMCs would require approximately 1000 l of culture volume using stirred-tank bioreactors, based on cell density data from our lab and the literature [1, 21, 28]. In contrast, the same production can be achieved with just 2 l of ColTubes. This dramatic reduction in culture volume not only makes large-scale production feasible and practical but also leads to substantial savings in labor, reagents, equipment, cGMP facility space, and overall manufacturing costs.

## 5. Conclusion

In conclusion, coltubes offers a unique combination of physiologically relevant culture microenvironment, exceptional performance, and scalability, making them a promising solution for addressing the challenges of large-scale cell manufacturing. Recent advancements in biology have provided efficient protocols for differentiating hPSCs into various human cell types [1, 47]. Future research can integrate these protocols with ColTubes to enable the production of diverse human cell types at a low cost. The presented work has demonstrated the potential of ColTubes using small scale cultures. Future work should focus on scaling up the cell culture using ColTubes (figure S5). To achieve that, specialized bioreactors for hosting ColTubes and novel medium feeding strategies should be developed.

## Data Availability

All data that support the findings of this study are included within the article (and any supplementary information files).
